# Paraneoplastic isolated adrenocorticotropic hormone deficiency revealed after immune checkpoint inhibitors therapy: new insights into anti-corticotroph antibody

**DOI:** 10.3389/fimmu.2023.1284301

**Published:** 2023-11-14

**Authors:** Shin Urai, Miki Watanabe, Hironori Bando, Yuma Motomura, Masaaki Yamamoto, Motoko Tachihara, Maki Kanzawa, Hidenori Fukuoka, Genzo Iguchi, Wataru Ogawa

**Affiliations:** ^1^ Division of Diabetes and Endocrinology, Department of Internal Medicine, Kobe University Graduate School of Medicine, Kobe, Japan; ^2^ Division of Diabetes and Endocrinology, Department of Internal Medicine, Kobe University Hospital, Kobe, Japan; ^3^ Division of Respiratory Medicine, Department of Internal Medicine, Kobe University Graduate School of Medicine, Kobe, Japan; ^4^ Division of Diagnostic Pathology, Kobe University Graduate School of Medicine, Kobe, Japan; ^5^ Medical Center for Student Health, Kobe University, Kobe, Japan; ^6^ Division of Biosignal Pathophysiology, Kobe University, Kobe, Japan

**Keywords:** ACTH, corticotroph, hypophysitis, paraneoplastic syndrome, irAE

## Abstract

**Introduction:**

A recently discovered facet of paraneoplastic adrenocorticotropic hormone (ACTH) deficiency exists in two forms: a paraneoplastic spontaneous isolated ACTH deficiency (IAD) and an immune checkpoint inhibitor (ICI)-related hypophysitis. Autoantibodies against corticotrophs, such as circulating anti-proopiomelanocortin (POMC) antibodies are considered disease markers. However, the number of identified cases was limited, implying that the characteristics of these autoantibodies are not fully understood.

**Methods:**

We investigate circulating autoimmune autoantibodies in detail through a novel case of IAD that developed as a paraneoplastic autoimmune ACTH deficiency.

**Results:**

The patient developed IAD after 25 weeks of ICI therapy for metastasis of large-cell neuroendocrine carcinoma at 69 years of age. Ectopic ACTH expression and infiltration of CD3+, CD4+, CD8+, and CD20+ lymphocytes were observed in the tumor tissues and circulating anti-POMC antibodies were detected specifically in the patient’s serum. Moreover, detailed analyses of immunofluorescence staining using patient serum revealed that the recognition site of the autoantibody was ACTH_25-39_, which had not been identified in previous cases of paraneoplastic autoimmune ACTH deficiency.

**Conclusion:**

This case involved a combination of paraneoplastic spontaneously acquired IAD and ICI-related hypophysitis occupying the middle ground. Moreover, our study reveals new aspects of anti-POMC antibodies in patients with paraneoplastic ACTH deficiency. This report expands our understanding of the immunological landscape and provides new insights for the identification of antibodies associated with paraneoplastic autoimmune ACTH deficiency.

## 1 Introduction

Hypophysitis is a rare inflammatory disorder involving the pituitary gland and infundibulum. It is classified into primary forms, encompassing autoimmune and other inflammatory or infiltrative manifestations, and secondary forms, which develop in response to local processes, systemic diseases, infections, neoplastic processes, or drug-induced conditions (1). The most common cause of hypophysitis is autoimmune disease, with adrenocorticotropic hormone (ACTH), the pituitary hormone, being the most affected in autoimmune hypophysitis (2). Adult-onset isolated ACTH deficiency (IAD) is a rare disease characterized by secondary adrenal insufficiency with low or absent cortisol production, normal secretion of other pituitary hormones, and no pituitary structural defects (3, 4). Some patients with IAD are assumed to have an autoimmune etiology similar to autoimmune hypophysitis (5, 6). Indeed, anti-pituitary antibodies (APA) such as anti-corticotroph antibodies, but not ACTH or other proopiomelanocortin (POMC)-derived peptides, have been detected in the serum of patients with IAD (7, 8). Moreover, autoimmune antibodies against S100β-positive cells have been detected in some patients with IAD, indicating the autoimmune involvement of follicular stellate cells (9). However, the role of autoimmune antibodies as fundamental disease markers of IAD has not been fully elucidated.

Paraneoplastic syndromes represent symptoms or manifestations resulting in organ or tissue damage distant from the neoplasm or its metastatic site, and can affect almost any organ or tissues (10). Paraneoplastic syndromes can also arise from autoimmunity to the components of particular organs, which is caused by the cross-reactivity of antitumor immunity (11).

Paraneoplastic autoimmune hypophysitis, a novel clinical condition, has been recently described with a common underlying mechanism, where ectopic expression of pituitary antigens in coexisting neoplasms triggers autoimmunity against pituitary-specific cells (1, 12). We have previously reported a case of spontaneous IAD onset before the diagnosis of large-cell neuroendocrine carcinoma (LCNEC) (13). The patient was diagnosed with LCNEC three years after the identification of the acquired IAD. Interestingly, the LCNEC tumor tissues exhibited ectopic ACTH expression, and an autoimmune antibody against POMC was identified in her serum. Additionally, immune checkpoint inhibitor (ICI)-related hypophysitis has been increasingly reported with the development of cancer immunotherapies (2). Ectopic ACTH expression in tumor tissues and circulating anti-POMC antibodies in the serum have also been identified in a subset of patients with ICI-related hypophysitis diagnosed with malignant melanoma or renal cell carcinoma (14).

Based on previous cases, paraneoplastic autoimmune ACTH deficiency has been shown to include the following two forms: paraneoplastic spontaneously acquired IAD and paraneoplastic ICI-related hypophysitis (15); however, the number of cases is still limited, and therefore, the details of these autoimmune antibodies as potential disease markers (e.g., anti-POMC antibody) remain unclear.

Here, we reveal a detailed site recognized by circulating autoimmune autoantibodies in the serum which had not been identified through a novel case of IAD with clinical features similar to those of two previously reported forms of paraneoplastic autoimmune ACTH deficiency.

## 2 Methods

### 2.1 Subjects and samples

This study was performed in accordance with the Declaration of Helsinki and approved by the Ethics Committee of Kobe University Graduate School of Medicine (Approval No.1685). All procedures were performed according to the guidelines of the approved protocol, and written informed consent was obtained from the patient. The patient consented to the submission to the journal.

Serum was collected from the patient with IAD at the time of diagnosis and frozen until use. Serum samples from healthy volunteers were also collected at the Kobe University Graduate School of Medicine.

### 2.2 Hormonal assays

The pituitary and peripheral hormone levels were measured to screen for hypopituitarism. The anterior pituitary function was assessed using insulin and thyrotropin-releasing hormone (16). Insulin-like growth factor-I (IGF-I) standard deviation score (SDS) was calculated based on age- and sex-matched healthy Japanese subjects, as previously reported (17).

### 2.3 DNA sequencing, analysis, and HLA typing

Genomic DNA was extracted from whole blood and formalin-fixed paraffin-embedded tissue specimens using the Gentra Puregene Blood Kit (QIAGEN, Hilden, Germany) and REPLI-g FFPE (QIAGEN), respectively. The *POMC* coding region was amplified from genomic DNA by polymerase chain reaction using the primers listed in **Supplementary Table 1**. *POMC* variants were analyzed by Sanger sequencing using forward and reverse primers as described above. Human leukocyte antigen (HLA) alleles were genotyped by next-generation sequencing using the MiSeq system (Illumina, RRID : SCR_016379) at the HLA Laboratory (Kyoto, Japan).

### 2.4 Immunohistochemical analyses in LCNEC tumors

Tissue specimens from the patient were fixed in 10% buffered formaldehyde, dehydrated in graded ethanol, and embedded in paraffin.

Immunostaining was performed using anti-CD56 (Cell Marque Cat# 156R-9, RRID : AB_2941091), anti-Ki67 (Agilent Cat# IR626, RRID : AB_2890068), anti-CD3 (Agilent Cat# M7254, RRID : AB_2631163), anti-CD4 (Roche Cat# 05552737001, RRID : AB_2335982), and anti-CD8 (Roche Cat# 05937248001, RRID : AB_2335985), and anti-CD20 antibodies (Agilent Cat# M0755, RRID : AB_2282030) using a BOND Max autostainer (Leica Microsystems, Wetzlar, Germany) according to the manufacturer’s protocols. The sections were stained with Hematoxylin and Eosin.

Immunostaining was also performed to detect ectopic ACTH expression in LCNEC tumors. After deparaffinization with xylene and graded ethanol, the tissues were subjected to antigen retrieval by autoclaving the sections in citric acid buffer (pH 6.0). Endogenous peroxidase activity was blocked with 3% hydrogen peroxide for 1 h. After blocking with 10% goat serum for 1  h, the specimens were incubated with anti-ACTH antibody (1:100, Abcam Cat# ab74976, RRID : AB_1280736) overnight at 4°C as a primary antibody. Tyramide signal amplification for immunofluorescence enhancement was performed according to the manufacturer’s protocol using an Alexa Fluor 488 Tyramide SuperBoost Kit (Thermo Fisher Scientific, Cat# B40922, RRID : AB_2941012). Nuclei were counterstained with Hoechst 33342 (Nacalai Tesque, Kyoto, Japan). All experiments were independently repeated at least three times and confirmed in different fields.

### 2.5 Immunofluorescence staining for revealing autoimmune antibody

Double immunofluorescence staining of mouse pituitary tissues was performed to investigate autoimmune antibodies against corticotrophs in the serum. Mouse pituitary tissue was fixed in 4% paraformaldehyde (Nacalai Tesque), dehydrated using a graded ethanol series, and embedded in paraffin. After deparaffinization, the tissues were subjected to antigen retrieval by autoclaving the sections in HistoVT One solution (Nacalai Tesque) at 90 °C for 20  min. Specimens were permeabilized for 45 min using phosphate-buffered saline supplemented with 0.3% Triton X-100 and blocked using a blocking one histo (Nacalai Tesque). Serum (1:50) and anti-ACTH antibodies (1:200, Abcam Cat# ab74976, RRID : AB_1280736) as primary antibodies using Can Get Signal immunostain Immunoreaction Enhancer Solution B (TOYOBO, Osaka, Japan) with 5% blocking agent were separately incubated overnight. Goat anti-human IgG (H+L) Alexa Fluor 488 (1:500, Thermo Fisher Scientific Cat# A-11013, RRID : AB_2534080) and donkey anti-rabbit IgG (H+L) Alexa Fluor 546 (1:500, Thermo Fisher Scientific Cat# A10040, RRID : AB_2534016) were used as secondary antibodies. Nuclei were counterstained with Hoechst 33342 (Nacalai Tesque).

Experiments were also performed to identify the site of corticotrophs that the autoantibodies recognized using a similar method, using serum pre-absorbed with recombinant proteins. For the pre-absorption test, serum samples (1:50) were incubated overnight at 4°C with or without the following recombinant human proteins: POMC (Abcam Cat# ab108118), ACTH_1-39_ (Abcam Cat# ab141151), ACTH_1-24_ (Abcam Cat# ab142251), and alpha-melanocyte-stimulating hormone (α-MSH) (Abcam Cat#ab120189) prior to immunofluorescence staining. All experiments were independently repeated at least three times.

Animal tissue experiments were performed in accordance with the guidelines of the Animal Ethics Committee of Kobe University Graduate School of Medicine. All protocols were approved by the Institutional Animal Care and Use Committee (P220605).

### 2.6 Images of immunohistochemical and immunofluorescence staining

All images were obtained using an All-in-one Fluorescence Microscope (BZ-X800 (Keyence, RRID : SCR_023617) or BZ-X700 (Keyence, RRID : SCR_016979) and reconstructed using the BZ-X Analyzer software (Keyence, Cat#BZ-H3A, RRID : SCR_017375). Data from a representative experiment are presented in the results section.

## 3 Results

### 3.1 Case presentation

We report a case involving a 69-year-old male patient diagnosed with secondary adrenal insufficiency who was referred to our department. He had a height of 172.7 cm and weighted 76.8 kg (body mass index: 25.7 kg/m^2^), with no previous history of autoimmune disease.

**Figure 1:**
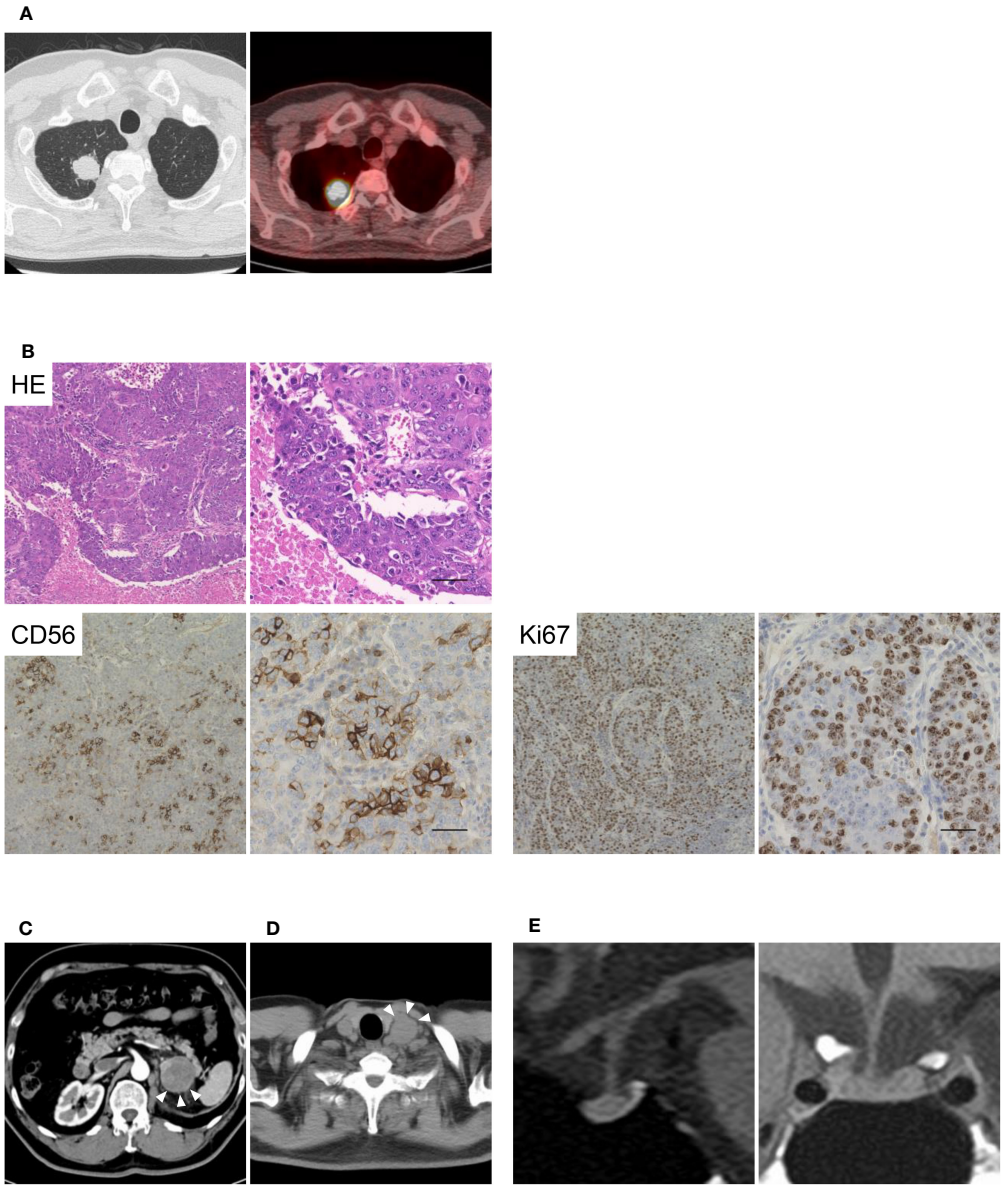
Images and pathological findings of the patient. The imaging and pathological diagnosis of large-cell neuroendocrine carcinoma (LCNEC) are shown in **Figure 1**. **(A)** Computed tomography and fluorine-18 fluorodeoxyglucose positron emission tomography of the lungs. **(B)** Histological findings of the LCNEC tissue at low and high magnifications. Hematoxylin and eosin staining, and anti-CD56 and anti-Ki67 staining are shown. Scale bar 50 µm. **(C, D)** Metastases of the left adrenal gland and left supraclavicular node are indicated by arrowheads. **(E)** T1-weighted magnetic resonance imaging (MRI) of the pituitary gland.

The patient was diagnosed with a pulmonary tumor at 62 years of age and underwent lung lobectomy (**Figure 1A**). As shown in **Figure 1B**, the specimens showed neuroendocrine morphology, such as trabeculae, palisading, and rosette formation, as well as cells positive for the neuroendocrine marker CD56, and a high proliferation rate; therefore, the tumor was diagnosed as LCNEC. Although the patient was treated with cisplatin and etoposide as adjuvant chemotherapy, the metastasis progressed to the left adrenal gland, and he underwent unilateral adrenalectomy at 63 years of age (**Figure 1C**). He received radiotherapy for para-aortic lymph node metastasis at the age of 67 years and left supraclavicular node metastasis at the age of 68 years (**Figure 1D**). However, radiation therapy failed to control the recurrent metastatic lesions, and combination immunotherapy with nivolumab (360 mg) and ipilimumab (1 mg/kg) was initiated. The endocrinological evaluation prior to initiating ICI treatment was almost normal, with a plasma ACTH level of 58.4 pg/mL, a cortisol level of 15.2 μg/dL, and no signs of adrenal insufficiency or Cushingoid. After ICIs therapy, the tumor shrank and remained in partial response. Therefore, combination immunotherapy was continued for 25 weeks until 69 years of age. There were no adverse events other than grade 1 pruritus according to the Common Terminology Criteria for Adverse Events Version 5.0 (18). In addition, no symptoms of adrenal insufficiency were observed, nor were there any decreases in plasma ACTH or cortisol levels.

Loss of appetite and general fatigue suddenly appeared 176 days after the initiation of ICI therapy, and the patient was admitted to our hospital. The patient was diagnosed with secondary adrenal insufficiency with a plasma ACTH 3.4 pg/mL and a cortisol 0.2 μg/dL with hyponatremia and eosinophilia. Endocrinological evaluation revealed that the basal ACTH and cortisol levels were extremely low, and the response to stimulating provocations was blunted; however, the secretion of other anterior pituitary hormones was not impaired (**Supplementary Table 2**). In addition, urinary free cortisol levels were below the detectable range. However, the use of exogenous glucocorticoids has not been recently reported. Magnetic resonance imaging performed 19 days after the onset of adrenal insufficiency revealed that the pituitary gland was almost normal in the absence of enlargement (**Figure 1E**). Therefore, the patient was diagnosed with IAD induced by treatment with ICIs and treated with hydrocortisone replacement. The patient resumed ICI therapy after hydrocortisone replacement and has not developed any other endocrine immune-related adverse events (irAEs) to date.

As shown in **Table 1**, the patient’s HLA alleles were HLA-DQA1*03:02 and DQB1*03:03, which were identified as susceptibility alleles for idiopathic IAD, as documented in a previous study (19). Additionally, he had HLA-DR15 encoded by HLA-DRB1*15:01, which is associated with ICI-related IAD (20, 21), and DR53 encoded by DRB4, which is associated with lymphocytic hypophysitis (22).

**Table 1:** HLA alleles.

HLA Class I		HLA Class II	
HLA-A	24:02	HLA-DRB1	09:01
	26:01		15:01
HLA-B	40:02	HLA-DRB4	01:03
	48:01	HLA-DRB5	01:01
HLA-C	03:04	HLA-DQA1	01:02
	08:03		03:02
		HLA-DQB1	03:03
			06:02
		HLA-DPA1	01:03
			02:02
		HLA-DPB1	02:01
			03:01

Human leukocyte antigen (HLA) alleles are shown. HLA alleles were genotyped by next-generation sequencing (NGS) using the MiSeq system (Illumina Inc. San Diego, CA, USA) at the HLA Laboratory (Kyoto, Japan).

### 3.2 Diagnosis of paraneoplastic autoimmune ACTH deficiency and analyses of autoimmune antibodies

Based on previous reports of paraneoplastic spontaneously acquired IAD with LCNEC and paraneoplastic ICI-related hypophysitis (13, 14), we hypothesized that the patient developed IAD as a form of paraneoplastic autoimmune ACTH deficiency after ICI treatment.

**Figure 2:**
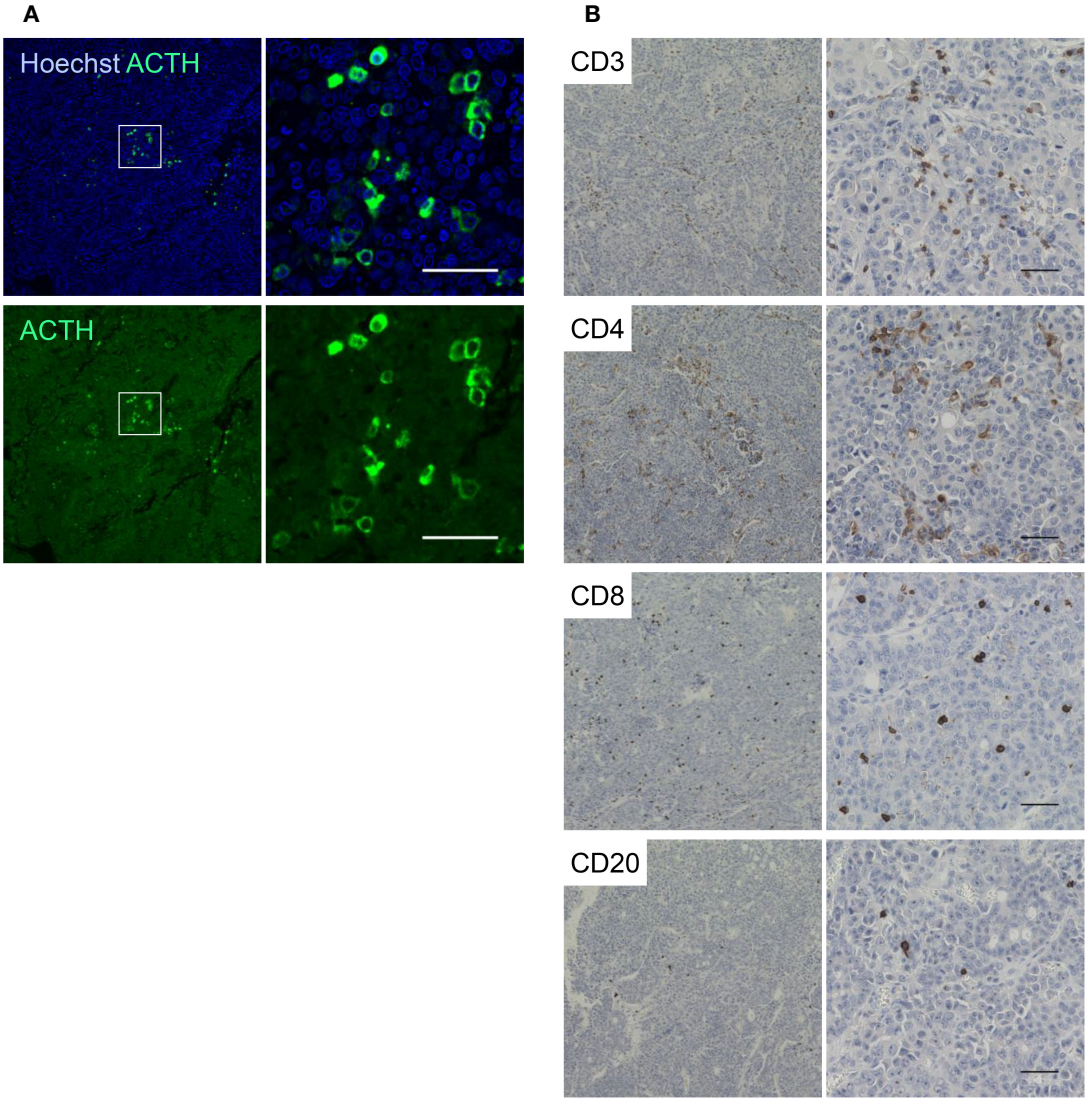
Ectopic ACTH expression in LCNEC tissues. **(A)** Ectopic expression of ACTH in LCNEC tissues as determined by immunohistochemistry. **(B)** Infiltration of CD3+, CD4+, CD8+, and CD20+ lymphocytes into the tumor. Representative images at low and high magnifications are shown. Scale bar 50 µm. ACTH, adrenocorticotropic hormone; LCNEC, large-cell neuroendocrine carcinoma.

We analyzed the ectopic expression of POMC in LCNEC tissues. No mutations in the tumor-derived genomic DNA were detected in the coding region of *POMC*. Interestingly, immunohistochemistry revealed ectopic ACTH expression in LCNEC tumor tissues, as shown in **Figure 2A**. Furthermore, in LCNEC tumor tissues, infiltration of CD3+, CD4+, and CD8+ lymphocytes as well as slightly CD20+ cells were observed, indicating tumor immunity and T cell involvement (**Figure 2B**).

**Figure 3:**
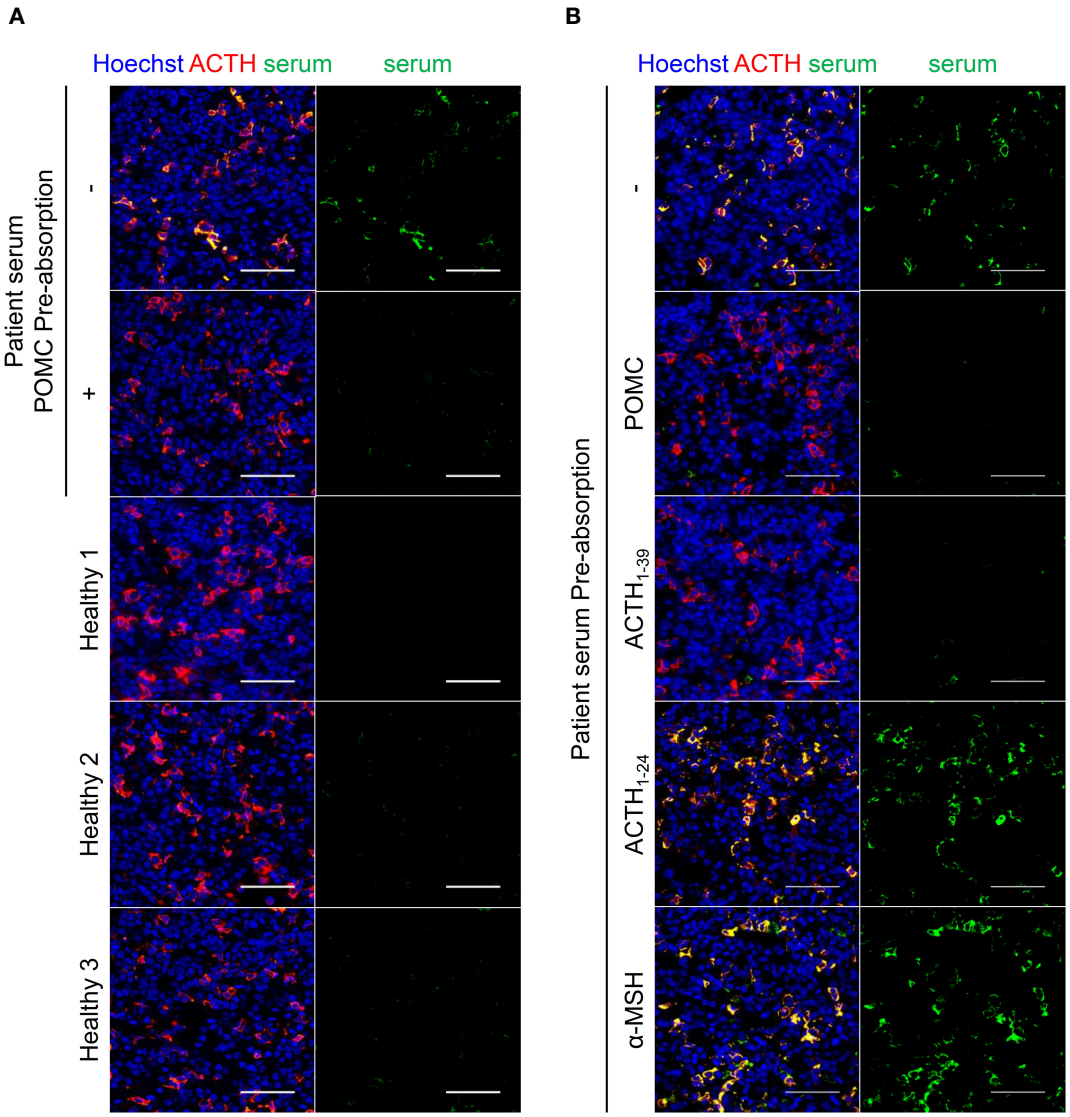
Immunofluorescence staining for detecting autoantibody. Immunofluorescence staining of patient serum and anti-ACTH antibody in the mouse pituitary. **(A)** Serum from the patient with specifically recognized corticotrophs compared with sera from healthy subjects. Pre-absorption of the patient’s serum with recombinant human POMC protein diminished reactivity to corticotrophs, indicating the presence of circulating anti-POMC antibodies in the serum. **(B)** Pre-absorption of POMC or ACTH_1-39_ protein into the serum diminished the signal, whereas pre-absorption of ACTH_1-24_ or α-MSH protein showed no signal reduction, indicating that ACTH_25-39_ was the region recognized by the circulating anti-POMC antibody. The representative results are presented in the figure. Scale bar 50 µm. ACTH, adrenocorticotropic hormone; POMC, proopiomelanocortin; α-MSH, alpha-melanocyte-stimulating hormone.

Next, we investigated the presence of autoimmune antibodies against corticotrophs in the serum. Immunofluorescence staining revealed an autoimmune antibody against the pituitary gland in the patient’s serum. As shown in **Figure 3A**, patient sera recognized the cytoplasmic proteins of mouse pituitary corticotrophs, whereas healthy sera did not. When the serum was pre-absorbed with recombinant POMC, the signals reacting with corticotrophs were reduced, demonstrating that this autoantibody selectively recognizes the POMC protein. Finally, we performed similar pre-absorption experiments with recombinant ACTH_1-39_, ACTH_1-24_, and α-MSH proteins as well as the POMC protein to identify the detailed location of POMC recognized by autoimmune antibodies in the patient’s serum. Intriguingly, the pre-absorption of ACTH_1-39_ protein into the serum, as well as POMC protein, diminished reactivity with corticotrophs, whereas the pre-absorption of ACTH_1-24_ or α-MSH proteins impaired reactivity reduction (**Figure 3B**). Thus, a circulating anti-corticotroph antibody was specifically detected in this patient compared to healthy subjects, suggesting that the sites of recognition for serum autoantibodies were ACTH_25-39_ within the POMC protein.

## 4 Discussion

Here, we report an exceptional case of IAD that manifested as a variant of paraneoplastic autoimmune hypophysitis. The patient was diagnosed with IAD post-ICI therapy and demonstrated ectopic ACTH expression in LCNEC tissues, concurrent with the presence of circulating anti-POMC antibodies. Furthermore, the immunoreactivity site of the serum autoantibody was identified as ACTH_25-39_, revealing previously uncharacterized aspects of the anti-POMC antibody in cases of paraneoplastic autoimmune ACTH deficiency.

This patient presented with paraneoplastic ICI-related IAD and was partially consistent with the previously reported clinical features of both paraneoplastic spontaneously acquired IAD and paraneoplastic ICI-related hypophysitis, occupying an intermediate position between these two forms. This case provides an expanded perspective on the pathology of paraneoplastic autoimmune hypophysitis.

Neuroendocrine differentiation in lung tumors has been reported over the years, and LCNEC is classified as a neuroendocrine carcinoma that confers the clinical phenotype of small-cell lung cancer (23). Ectopic ACTH syndrome (EAS), also referred to as ectopic Cushing’s syndrome, is a rare but life-threatening disease frequently associated with neuroendocrine tumors, including small cell lung cancer and bronchial carcinoids (24). Typical paraneoplastic endocrine syndromes, such as EAS, are directly caused by the ectopic production of hormones and bioactive peptides in tumors, whereas paraneoplastic autoimmune endocrine disorders are immune-mediated, which may be caused by HLA restriction and thus appear to be extremely rare (15, 25). Moreover, not all patients with paraneoplastic syndromes have detectable antibodies in their sera (10). Many patients with autoimmune-related paraneoplastic syndromes have cross-reactive antibodies in their sera between normal tissues and underlying neoplasms, which are commonly associated with paraneoplastic neurological syndromes (10, 15).

Interestingly, the most frequently ectopically expressed pituitary hormone in tumors is ACTH, which is observed in patients with tumors without hormone production as well as in EAS. Indeed, POMC is expressed in tumor tissues in 48% of patients with non-small cell lung cancer, with only a few cases manifesting as EAS with excess cortisol (26). Similarly, silent POMC expression has been observed in carcinoid tumors without EAS (27). Although the POMC processing pathway to produce ACTH is complicated by the requirement of prohormone convertases and mature secretory vesicles, some non-pituitary tumors may not be sufficiently differentiated to generate this pathway (28). It is hypothesized that ectopic ACTH expression is on two sides of the same coin and, if minimal, may cause immune reactivity as opposed to hormonal excess (29).

As previously reported, in a case of paraneoplastic spontaneously acquired IAD with LCNEC, corticotroph-reactive cytotoxic T cells (CTLs) were implicated as the etiologic agent and autoantibodies against corticotrophs were disease markers that were not pathogenic (13). The present research has not explored the existence of CTLs against corticotrophs in the pituitary gland, because it was not feasible to obtain primary peripheral mononuclear cells and pieces of pituitary tissue. Therefore, additional studies are necessary to advance our understanding of the mechanisms leading to pituitary injury due to corticotroph-reactive CTLs, as well as the potential adverse effects of autoimmune antibodies.

In the present analysis, the location of the POMC recognized by autoimmune antibodies in the patient’s serum was ACTH_25-39_. POMC, a polypeptide precursor of ACTH, is present in various tissues, including the pituitary gland, hypothalamus, central nervous system and skin (30). ACTH_1-24_ has steroidogenic activity, whereas ACTH_22-39_ is less steroidogenic and has higher immunological activity than ACTH_1-24_ (31). Thus, it seems reasonable to identify ACTH_25-39_ as the region recognized by the circulating anti-POMC antibody. Circulating anti-POMC antibodies in the serum were identified both in a previously reported case of spontaneous IAD with LCNEC and in patients who had ICI-related hypophysitis diagnosed with malignant melanoma or renal cell carcinoma (13, 14). However, the specific recognition site of these antibodies remained undetermined. In this study, the recognition site of anti-POMC antibodies, ACTH_25-39_, was clarified in more detail. However, further investigation is necessary to determine whether the location of the POMC recognized by autoimmune antibodies in the patient’s serum is consistent with that of other cases of paraneoplastic ACTH deficiency and ICI-related hypophysitis. In addition, more cases are required to confirm the identification of this immunological site and to clarify the significance of these antibodies.

Circulating APA have been detected in several pituitary diseases and are surrogate markers of pituitary autoimmunity (32). APA measurement using immunofluorescence could be useful for the early prediction of anterior pituitary dysfunction (33, 34). In addition, endocrine-specific autoantibodies have been reported to aid in the early identification of ICI-induced endocrine disruptions and serve as predictive markers for treatment (35). Anti-POMC antibodies have recently been identified, and as a result, the utility for screening for these antibodies requires further studies. The measurement of anti-POMC antibodies may facilitate the screening of paraneoplastic autoimmune ACTH deficiency, especially in patients treated with ICIs. This is because the incidence of irAEs, including endocrine ones, has drastically increased with the widespread use of ICI therapy for various cancers. The symptoms of adrenal insufficiency closely resemble those associated with malignancies, making it difficult to diagnose IAD, particularly in patients with cancer. In certain cases, the condition may go unnoticed, or the diagnosis may be delayed. Therefore, further studies are needed to clarify whether the identification of the specific site of the anti-POMC antibodies in the present analyses can facilitate the early detection of paraneoplastic autoimmune ACTH deficiency.

Our study has some limitations, and several unresolved issues remain. Although the patient’s serum was collected at the time of IAD diagnosis, no samples were available before ICI administration, leaving undetermined trends in antibody titers before and after ICI therapy. Moreover, the sensitivity of anti-pituitary antibody detection may have been marginally diminished owing to the utilization of mouse pituitary tissue. Further studies using comprehensive models are required to elucidate the intricate pathogenesis of paraneoplastic autoimmune hypophysitis.

In conclusion, we presented a novel case of paraneoplastic ICI-related IAD and revealed that the immunoreactivity site of the serum autoantibody was ACTH_25-39_ within POMC. This report expands our understanding of the immunological landscape and provided new insights into the identification of circulating autoimmune antibodies in patients with paraneoplastic autoimmune ACTH deficiency as a form of paraneoplastic autoimmune hypophysitis.

## Data availability statement

Original datasets are available in a publicly accessible repository: The original contributions presented in the study are publicly available. This data can be found here: GenBank DNA Data Bank of Japan (DDBJ) under the accession number LC778517.

## Ethics statement

The studies involving humans were performed in accordance with the Declaration of Helsinki and approved by the Ethics Committee of Kobe University Graduate School of Medicine (Approval No.1685). All procedures were performed according to the guidelines of the approved protocol. The studies were conducted in accordance with the local legislation and institutional requirements. The participants provided their written informed consent to participate in this study. Animal tissue experiments were performed in accordance with the guidelines of the Animal Ethics Committee of Kobe University Graduate School of Medicine. All protocols were approved by the Institutional Animal Care and Use Committee (P220605). The study was conducted in accordance with the local legislation and institutional requirements.

## Author contributions

SU: Conceptualization, Data curation, Formal Analysis, Investigation, Methodology, Writing – original draft. MW: Data curation, Investigation, Writing – original draft. HB: Conceptualization, Funding acquisition, Investigation, Methodology, Project administration, Writing – review & editing. YM: Data curation, Writing – review & editing. MY: Formal Analysis, Writing – review & editing. MT: Data curation, Writing – review & editing. MK: Investigation, Writing – review & editing. HF: Writing – review & editing, Data curation. GI: Conceptualization, Funding acquisition, Investigation, Methodology, Project administration, Supervision, Writing – review & editing. WO: Supervision, Writing – review & editing.
