# Supplementary material for: Paraneoplastic isolated adrenocorticotropic hormone deficiency revealed after immune checkpoint inhibitors therapy: new insights into anti-corticotroph antibody
Source: Front Immunol. 2023 Nov 14;14:1284301. doi: 10.3389/fimmu.2023.1284301 (PMC10682701; doi:10.3389/fimmu.2023.1284301)
Supplement: Supplementary file 1 [file Table_1.docx]

Supplementary Material

# Supplementary Table.1 Primers for PCR to analyze the *POMC* gene.

|  | Forward (5’ – 3’) | Reverse (5’ – 3’) |
| --- | --- | --- |
| Exon1 | GCCGGGAAGGTCAAAGTC | ATCCCGGGGAAAGAGCAC |
| Exon2 | CCCACCAATCTTGTTTGCTT | TGTCTAAGCCAAGATGGCAGT |
| Exon3 | CTCATGCCCTCGCGTCTT | AGACGTCCTCGCGCTTCT |
|  | AGTACGTCATGGGCCACTTC | TCAGCTCCCTCTTGAACTCC |
|  | GCGCCCAGTGAAGGTGTA | GTCTGGCTCTTCTCGGAGGT |
|  | CCCCTACAGGATGGAGCACT | GGCAGCTTTAAGAGGCTGATT |

The proopiomelanocortin (*POMC*) coding region was amplified from genomic DNA via polymerase chain reaction (PCR) and analyzed by Sanger sequencing using the primers listed in table.

# Supplementary Table.2 Endocrinological data

|  |  | Results | Reference range |
| --- | --- | --- | --- |
| ACTH (pg/mL) | basal | 2.7 | 7.7 – 63.1 |
|  | peak | 2.3 |  |
| cortisol (μg/dL) | basal | <0.2 |  |
|  | peak | 0.2 |  |
| GH (ng/mL) | basal | 0.20 | <3 |
|  | peak | 12.64 |  |
| PRL (ng/mL) | basal | 17.8 | 3.0 – 17.3 |
|  | peak | 81.0 |  |
| TSH (μIU/mL) | basal | 3.85 | 0.610 – 4.230 |
|  | peak | 22.50 |  |
| UFC (μg/day) |  | <3.2 | 5.5 – 66.7 |
| IGF-I (ng/mL) |  | 122 |  |
| IGF-I SDS |  | -0.1 |  |
| free T4 (ng/dL) |  | 1.29 | 0.90 – 1.70 |
| FSH (mIU/mL) |  | 23.6 | 1.8 – 12.0 |
| LH (mIU/mL) |  | 17.2 | 2.2 – 8.4 |
| T (ng/mL) |  | 4.31 | 1.31 – 8.71 |

Endocrinological data were also presented. Stimulation tests were performed using insulin (0.1 unit/kg) and thyrotropin-releasing hormone (200 μg) to assess anterior pituitary function.

ACTH, adrenocorticotropic hormone; GH, growth hormone; PRL, prolactin; TSH, thyroid-stimulating hormone; UFC, urine-free cortisol; IGF-I, insulin-like growth factor-I; free T4, free thyroxine; FSH, follicle-stimulating hormone; LH, luteinizing hormone; T, testosterone.
